# Not All Arms of IgM Are Equal: Following Hinge-Directed Cleavage by Online Native SEC-Orbitrap-Based CDMS

**DOI:** 10.1021/jasms.4c00094

**Published:** 2024-05-20

**Authors:** Victor Yin, Evolène Deslignière, Nadia Mokiem, Inge Gazi, Rolf Lood, Carla J. C. de Haas, Suzan H. M. Rooijakkers, Albert J. R. Heck

**Affiliations:** † Biomolecular Mass Spectrometry and Proteomics, Bijvoet Center for Biomolecular Research and Utrecht Institute for Pharmaceutical Sciences, Utrecht University, Padualaan 8, 3584 CH Utrecht, The Netherlands; ‡ Netherlands Proteomics Center, Padualaan 8, 3584 CH Utrecht, The Netherlands; § Genovis AB, Scheelevägen 2, 223 63 Lund, Sweden; ∥ Department of Medical Microbiology, University Medical Center Utrecht, Utrecht University, 3584 CX Utrecht, The Netherlands

## Abstract

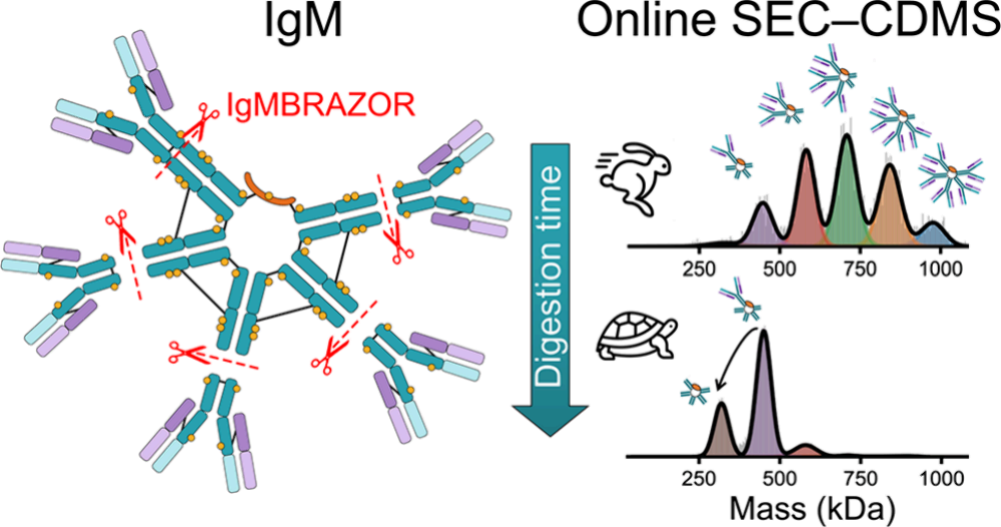

Immunoglobulins M (IgM) are key natural antibodies produced initially in humoral immune response. Due to their large molecular weights and extensive glycosylation loads, IgMs represent a challenging target for conventional mass analysis. Charge detection mass spectrometry (CDMS) may provide a unique approach to tackle heterogeneous IgM assemblies, although this technique can be quite laborious and technically challenging. Here, we describe the use of online size exclusion chromatography (SEC) to automate buffer exchange and sample introduction, and demonstrate its adaptability with Orbitrap-based CDMS. We discuss optimal experimental parameters for online SEC-CDMS experiments, including ion activation, choice of column, and resolution. Using this approach, CDMS histograms containing hundreds of individual ion signals can be obtained in as little as 5 min from single injections of <1 μg of sample. To demonstrate the unique utility of online SEC-CDMS, we performed real-time kinetic monitoring of pentameric IgM digestion by the protease IgMBRAZOR, which cleaves specifically in the hinge region of IgM. Several digestion intermediates corresponding to processive losses of F(ab’)_2_ subunits could be mass-resolved and identified by SEC-CDMS. Interestingly, we find that for the J-chain linked IgM pentamer, cleavage of one of the F(ab’)_2_ subunits is much slower than the other four F(ab’)_2_ subunits, which we attribute to the symmetry-breaking interactions of the J-chain within the pentameric IgM structure. The online SEC-CDMS methodologies described here open new avenues into the higher throughput automated analysis of heterogeneous, high-mass protein assemblies by CDMS.

## Introduction

**Figure 1:**
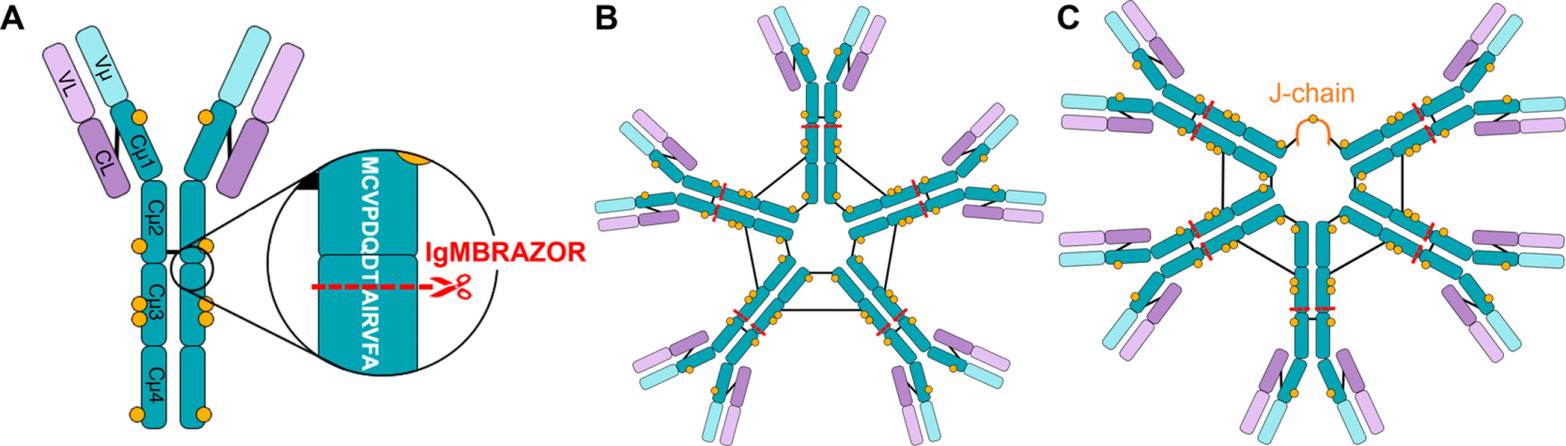
Structural details of IgM. (A) The IgM protomer resembles somewhat the IgA protomer, although it is longer. The μ heavy chain is ∼576 amino acids long and includes a variable domain (Vμ ∼ 110 amino acids, represented in light blue), four distinct constant region domains (Cμ1, Cμ2, Cμ3, Cμ4, each ∼110 amino acids, depicted in dark blue), and a “tailpiece” of ∼20 amino acids. Each protomer contains 10 N-glycosylation sites (indicated with orange dots). The μ chains in each monomer are covalently linked with a disulfide bond at Cys337 (black line). Each light chain (variable domain VL = light purple, constant domain CL = dark purple) is disulfide bonded to the μ chain using Cys136 in the μ chain. IgMBRAZOR cuts specifically just below Cys337, between Thr343 and Ala344, in the stretch (...VPDQDT/AIRVFA...) (red dashed line). (B) Initially it was thought that IgM protomers form highly symmetric, starfish-like pentamers with C5 symmetry. The five IgM protomers are stabilized by interprotomer disulfide bridges (black lines). (C) Representative schematic of the textbook structure of J-chain coupled IgM. In this structure the C5 symmetry is reduced to C2 symmetry, as only two of the protomers are bound to the linking J-chain. Moreover, there is a wide gap between the two latter protomers, that can accommodate the AIM/CD5L protein. Whether this molecule still contains true C2 symmetry is debatable, as this requires the J-chain to be both symmetric and evenly localized in the gap.

Immunoglobulin M (IgM) is the first antibody secreted by the adaptive immune system in response to a foreign antigen, and is the most potent inducer of the classical activation pathway of the complement system.^1,2^ IgM also regulates the immune tolerance and maintains homeostasis through the recognition and clearance of apoptotic cells and cellular debris.^3,4^ In contrast to IgG, IgM is oligomeric and usually consists of five IgM protomers (Figure 1A), enabling IgM to bind in theory up to ten antigens. In the early days, primarily based on negative-stain electron microscopy (EM) images, it was proposed that IgM exhibits a starfish-shaped, highly symmetric pentagonal structure with C5 symmetry (Figure 1B).^5,6^ However, under normal physiological conditions IgM is secreted into the bloodstream as a J-chain coupled pentamer.^5,7^ In these structures IgM is stabilized not only by covalent disulfide bonds between the Fc regions of the protomers, but also by disulfide bonds between the C-termini of just two Fc arms and the joining J-chain (Figure 1C).^8^ The incorporation of the J-chain breaks the C5 symmetry, making the J-chain-containing IgM pentamer an asymmetric pentagon with an open groove.^9^ In serum this groove was shown to accommodate the AIM/CD5L protein.^9,10^ When produced recombinantly, IgM can assemble independently of the J-chain, forming mixtures of primarily tetra-, penta- and hexamers. Such J-chain devoid IgM oligomers are sometimes also observed in circulation, albeit often at much lower concentration, at least under normal physiological conditions.^11^ Unless otherwise explicitly noted, we will use the term “IgM” to refer strictly to the pentameric, J-chain-containing IgM structure (Figure 1C).

IgM possesses five putative N-glycosylation sites on each Fc chain^12^ (Figure 1), leading to 10 per protomer and 51 N-glycosylation sites in total, including one N-glycosylation site on the J-chain. These glycans contribute substantially to the heterogeneity and mass of IgM, which exhibits a cumulative total molecular weight of ∼950 kDa. Given its crucial role in immune defense, and the emerging role of IgM as a potential biotherapeutic, there has been a renewed interest in studying IgM molecules by mass spectrometry (MS) in recent years.^3,13,14^

Over the past decades, native MS has established itself as a key analytical technique for structural biology, including several important biopharmaceutical applications.^15−22^ Native MS analyzes intact proteins and their noncovalent complexes while preserving native structural features, enabling characterization of their composition and stoichiometry.^23−25^ For these electrospray-based MS experiments, masses are not measured directly, but are inferred from the mass-to-charge (*m*/*z*) ratios of ensemble measurements of millions of charge-resolved ions. Such resolved charge state distributions are typically obtained easily for mass-homogeneous proteins. However, for analytes that are very large or polydisperse, overlap between charge states can create unresolvable mass profiles from which individual charges states cannot be extracted, hampering mass determination. Being the largest and most glycosylated antibody in humans, IgM falls into the category where conventional native MS typically fails. Recently, Orbitrap-based charge detection MS (CDMS) has been proposed as a strategy to overcome some of the limitations of ensemble native MS, by analyzing single ions instead of ion clouds.^26−28^ CDMS simultaneously measures the charge and the *m*/*z* ratio of each ion, allowing individual masses to be calculated without relying on charge state inferencing. This approach has proved to be extremely powerful for high mass and/or heavily glycosylated analytes.^29^ Illustratively, our group demonstrated earlier how the additional charge dimension offered by Orbitrap-based CDMS can resolve distinct oligomers formed when IgM is recombinantly produced without a J-chain.^10,26^

In the present work, we take the structural characterization of IgM one step further, and investigate in detail the enzymatic degradation of IgM by IgMBRAZOR, a novel protease which specifically cleaves human IgM at a single sequence site, liberating F(ab’)_2_ moieties (Figure 1A). Our ultimate aim was to sample the reaction and characterize its kinetic properties under physiological (*i.e*. buffered^30^) conditions. To achieve this, we adapted and combined online size exclusion chromatography (SEC)^31−33^ with Orbitrap-based CDMS to deal with the size and heterogeneity of all co-occurring IgM subunits, while simultaneously enhancing analysis throughput by automating rapid online buffer exchange. Recent developments in SEC columns allow drastic reductions in analysis times (<5 min.).^34^ The online coupling of SEC to single molecule CDMS (SEC-CDMS) is *a priori* not expected to be straightforward because of substantial variation in ion flux along the elution, making it difficult to maintain the ultralow ion population regime needed for CDMS. In addition, because SEC elution windows only span tens of seconds, scan numbers are limited, thereby reducing achievable ion statistics. Notwithstanding these obstacles, Bones et al. recently demonstrated the feasibility of SEC-CDMS using the manufacturer-supported Direct Mass Technology (DMT) platform.^35^ This first proof-of-concept study showed that accurate masses, in line with static infusion data, could be obtained for proteins up to 466 kDa.

Here, we build further on this work, and by using IgM as a model system, we report on the optimization of experimental parameters that must be considered for a successful online SEC-CDMS experiment, while also clearly describing several encountered practical pitfalls. The automated SEC-CDMS setup enabled real-time monitoring of the IgM digestion into F(ab’)_2_ moieties and the remaining pentamer Fc-core, with accurate mass identification of all co-occurring products formed over time. Surprisingly, we observed that in the digestion of IgM by IgMBRAZOR, the rate of release of F(ab’)_2_ arms is comparable for 4 out of 5 arms, but much slower for the “final” fifth F(ab’)_2_ arm. Our studies bolster earlier propositions, made on the basis of high-resolution experimental structures, that the symmetry within J-chain-containing IgM pentamers is broken by the asymmetric proximity of the J-chain to one of the protomers. Overall, we show that SEC-CDMS allows to characterize heterogeneous and large proteins in a single run, enabling unique experimental designs such as monitoring of reaction kinetics from physiological buffers.

## Experimental Section

### Materials

Ammonium acetate was obtained from Sigma-Aldrich (A2706). Phosphate-buffered saline (PBS) was obtained from Capricorn Scientific (PBS-1A). IgMBRAZOR was sourced from Genovis (Kävlinge, Sweden). XT sample buffer, 12 + 2-well 4–12% Criterion XT Bis-Tris Precast Gel, XT MOPS running buffer and Precision Plus Protein Dual Color Standards used for sodium-dodecyl sulfate–polyacrylamide gel electrophoresis (SDS-PAGE) were purchased from Bio-Rad (Veenendaal, The Netherlands). Imperial Protein Stain (24615) was sourced from Thermo Scientific, Rockford, USA. Milli-Q ultrapure water was used for all mobile phases and solutions unless otherwise noted.

### Production of IgM Constructs

Recombinant anti-StrepTag IgM with or without J-chain were produced as extensively described by Muts et al.^36^ In short, the variable heavy and light chains of anti-StrepTagII IgG (WO 2015/067768 A1, 2015) with upstream KOZAK and HAVT20 signal peptide were cloned into adapted pcDNA34 vectors (ThermoFisher Scientific), upstream the IgM heavy and kappa light chain constant regions.^37^ A plasmid coding for J-chain expression was kindly provided by Theo Rispens. After sequencing, plasmids were used to transfect EXPI293F cells (ThermoFisher Scientific), grown in EXPI293 medium at 37 °C, 8% CO_2_. For transfection, 1 μg DNA/mL cells (ratio of heavy and light chain plasmids is 2:3 for IgM). For expressions of IgM containing the J-chain, the J-chain plasmid was used as 50% of total plasmid. After 5 days of expression, the cell supernatant was collected by centrifugation and filtration (0.45 μm) and subsequently dialyzed against PBS. After dialysis, recombinant IgM was purified using POROS CaptureSelect IgM Affinity matrix column and dialyzed against PBS500. Finally, IgM with or without J-chain was further purified via SEC using a Superose 6 Increase 10/300 GL (≥95% purity).

### Following IgMBRAZOR-Induced IgM Degradation by SDS-PAGE

Amounts of 6 μg IgM were incubated with 0.025, 0.1, 0.25, and 12.5 U IgMBRAZOR for 30 min at ambient temperature. The reaction was stopped by mixing the sample with XT sample buffer at a ratio of 3:1 (v/v) and incubating the mixture at 50 °C for 5 min. IgM control (6 μg undigested IgM) and protease control (12.5 U IgMBRAZOR) samples were similarly treated with XT sample buffer. All samples were loaded onto a 12 + 2-well 4–12% Criterion XT Bis-Tris Precast Gel, and electrophoresis was run in XT MOPS running buffer for 10 min at 80 V, followed by 45 min at 200 V, until the dye front reached the bottom of the gel. Precision Plus Protein Dual Color Standards were run on the gel in parallel with the samples for protein size reference. The gel was stained for 1 h with 25 mL Imperial Protein Stain, followed by overnight destaining in ultrapure Milli-Q water. The resulting gel was scanned with an Amersham Imager 600 (GE Healthcare Life Sciences, Chicago, USA).

### Sample Preparation for SEC-MS

For measurements without IgMBRAZOR, IgM samples were measured without further preparation. For measurements of fully digested IgM, 50 U of IgMBRAZOR was added to a 100 μL solution of 0.25 μg/μL IgM and incubated at ambient temperature for 30 min prior to analysis. For kinetic measurements using IgMBRAZOR, separate solutions of IgM and protease were first prepared in PBS (total volume approximately 100 μL). Both solutions were then thermally pre-equilibrated by storage in an Agilent 1290 Infinity autosampler chamber set to 7 °C. Immediately prior to acquisition, the two solutions were mixed by repetitive pipetting, and instrument acquisition initiated. The final concentration of IgM was 0.25 μg/μL, with variable levels of IgMBRAZOR as described in the main text. Subsequent reaction time points were collected by repeatedly sampling the reaction mixture stored in the autosampler chamber.

### Online Native SEC-MS

Native SEC analysis was performed using an Agilent 1290 Infinity HPLC system, equipped with either a NativePac OBE-1 SEC column (2.1 × 50 mm, 3 μm particle size, 80 Å; Thermo Fisher) or an ACQUITY UPLC Protein BEH SEC 200 Å Column (4.6 × 300 mm, 1.7 μm particle size; Waters), using isocratic 100 mM ammonium acetate pH 6.8 as mobile phase. A tabulation of flow rates is included in Table S1. Following column elution, the flow was split in a 1:65 ratio toward the MS and an Agilent 1200 Infinity variable wavelength detector set at 280 nm, respectively.

All MS experiments were performed on a Q Exactive UHMR Orbitrap mass spectrometer (Thermo Scientific, Bremen, Germany) equipped with an ECD cell (e-MSion, Corvallis, USA), which was set in transmission-only mode for all measurements. The SEC-LC was interfaced to the MS via a Nanospray Flex ESI source, with a nanobore stainless steel emitter (Thermo Fisher) operated in positive ion mode with a spray voltage of 2.7 kV. The source temperature was set at 250 °C. Nitrogen was used as collision gas. Ion injection times (typically around 100 ms) were optimized for each set of experiments to maintain single ions levels (below ∼ hundreds of ions per scan) over the chromatographic elution time. All samples of IgM for SEC-MS analysis were prepared to a final concentration of 0.25 μg/μL using PBS buffer. Samples were loaded onto a 96-well twin.tec PCR LoBind plate (Eppendorf) for analysis. A sample amount of 0.5 μg was injected for each measurement. A tabulation of key MS instrument parameters can be found in Table S1. To maintain a quantitative, linear relationship between single ion intensities and charge, the instrument noise threshold was set to 0, automatic gain control (AGC) was set to fixed, and a *m*/*z* scan range of 5,000–20,000 was utilized to prevent inadvertent injection time modifications.

### MS Data Processing and Analysis

Single particle Orbitrap-based CDMS data was processed in Python as previously described,^26^ with minor modifications to facilitate processing of SEC-CDMS data. In brief, only frequency-domain data (*i.e*. the final mass spectrum per scan) were used for single ion analysis. Single ion intensities were corrected for injection time normalization. A fwhm filtering value of 3 was utilized. Only MS scans prior to salt elution were utilized for analysis. An intensity-to-charge calibration factor of 14.401 was used for all measurements. SciPy was used for Gaussian fitting of mass histograms.^38^

## Results and Discussion

### Orbitrap-Based CDMS at Chromatographic Time Scales – Ion Flux Considerations

Orbitrap-based CDMS, representing a single ion measurement technique, relies on the acquisition of MS scans that contain at most one ion in a given *m*/*z* window.^26,27^ This requirement, at a first glance, would render Orbitrap-based CDMS incompatible with chromatographic hyphenation due to analyte concentrations strongly fluctuating during the elution. Depending on how ion transmission is optimized, single ion MS scans during an elution event would be expected to either (1) under-sample the leading and trailing edges of the chromatographic peak, or (2) oversaturate at the apex of the peak. Both scenarios would lead to substantial decreases in the quantity of usable scans in CDMS. One way to tackle this issue is to use the Automatic Ion Control (AIC) method included in the manufacturer’s version of Orbitrap-based CDMS (*i.e*., Direct Mass Technology), whereby ion injection times are adjusted on-the-fly to maintain single ion levels across changing analyte concentrations.^39^

Here, we hypothesized that deliberately controlling the ion flux may not be necessary for online SEC-CDMS measurements. We reasoned that as long as any new ion signals emerging from the increasing analyte concentration appear in sufficiently different *m*/*z* windows to not overlap, the intrinsic ability of the Orbitrap mass analyzer to accommodate multiple individual ion signals (*i.e*. multiplexing^27^) could be exploited, allowing the accumulation of ∼ hundreds of different ion signals in an individual MS scan without departing the essential single ion regime. Fortuitously, Orbitrap-based native CDMS is frequently used to analyze highly heterogeneous analytes that lack well-resolved charge states and are thus not amenable to standard ensemble native MS.^29^ For these analytes, which by definition do not have their signals well-concentrated at particular *m*/*z* values, this intrinsic signal dispersion may render them particularly amenable to online LC-CDMS analysis. Due to their large molecular weight and glycosylation-induced mass heterogeneity, IgMs exhibit ideal properties to test such an approach.

**Figure 2:**
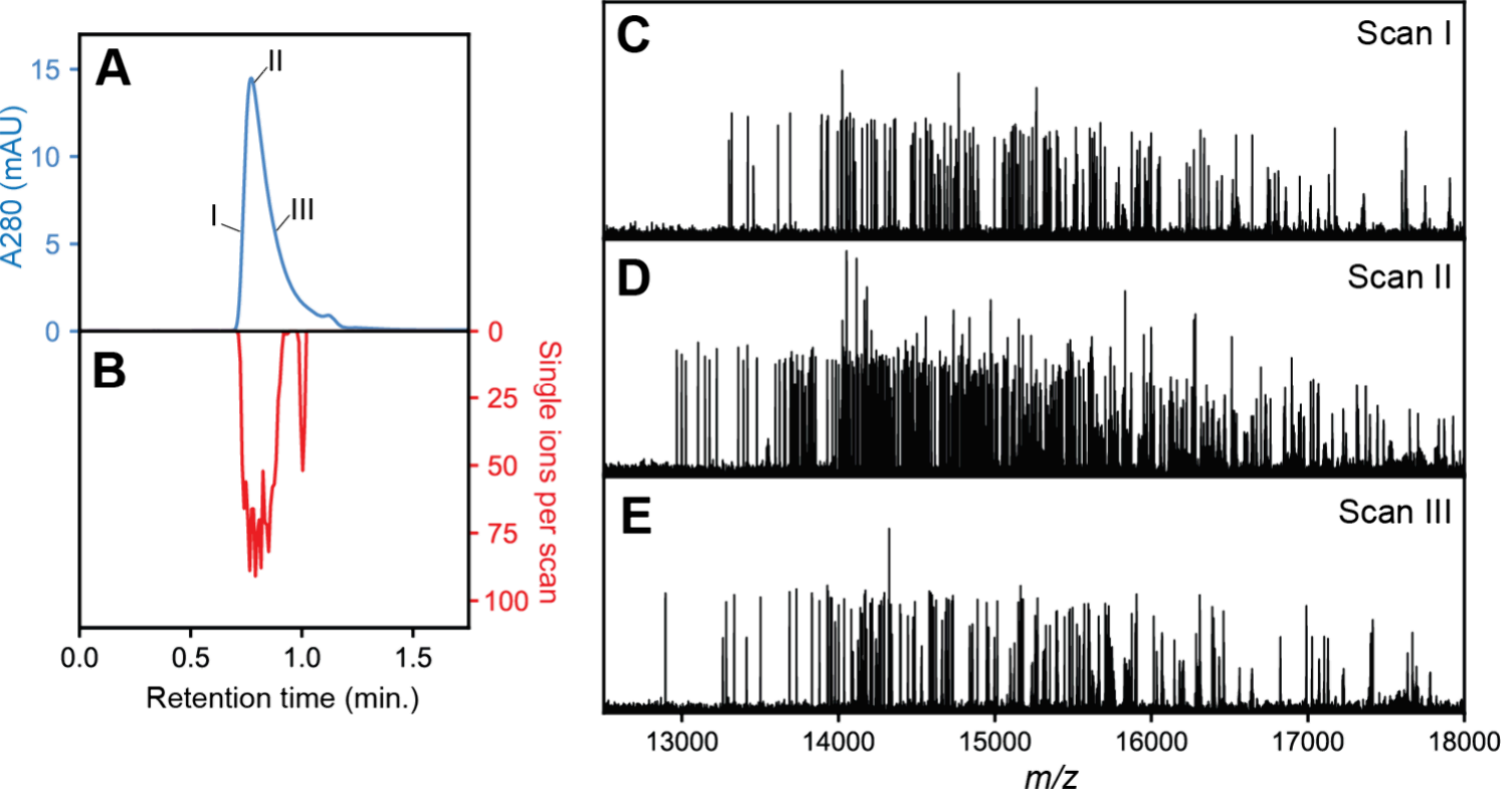
Generation of single ion regime OBE-CDMS scans of IgM using fixed ion injection times. (A) UV chromatogram from OBE-CDMS analysis of IgM. Representative single ion scans were taken from the annotated positions, labeled I–III. (B) Number of single ions extracted per MS scan as a function of retention time over the elution profile. The length of the transient per MS scan was set to 512 ms, corresponding to a resolution setting of 100,000. (C–E) Representative MS scans extracted from positions I–III at a fixed ion injection time of 100 ms. Despite the change in ion flux, distinct single ion “shelves” can be observed in all scans, without substantial numbers of ions co-occurring in the same *m*/*z* window.

To demonstrate the validity of this hypothesis, we chose to initially use the NativePAC OBE-1 column (hereby referred to as OBE). This is a short SEC column purposely designed for rapid buffer exchange and subsequent elution with very fast runtimes (<5 min), and thus not well-suited for analyte separation.^40^ Because of the very narrow chromatographic peaks arising from the OBE column, it represents a likely “worst case scenario” with respect to rapidly changing ion flux and its associated challenges. We tested OBE-CDMS using a fixed ion injection time to assess whether single ion scans can be maintained over the short chromatographic elution (Figure 2). Optimization of MS parameters for single ion measurements was performed as previously described,^29^ and a tabulation of experimental parameters can be found in Table S1. Low ion count MS scans, enabling single ion monitoring, could indeed be maintained under these conditions (Figure 2**C-E)**. Even at the elution apex, where the analyte concentration is highest (Figure 2D), excess single ions are still accommodated by their separation into different *m*/*z* windows. Therefore, at least for highly heterogeneous analytes, these SEC-CDMS-derived signals should remain fully amenable to analysis without requiring any real-time ion injection time adjustment.

### Maximizing Single Ion Signals during the OBE Chromatographic Elution Window

The short elution profile of the OBE column (approximately 15 s) severely limits the time during which the analyte signal is available for CDMS analyses.^32^ This time regime is more than an order of magnitude shorter than acquisition times for static-spray CDMS experiments, which typically last several minutes.^41^ In an ideal scenario, CDMS histograms would be capable of being generated from a single injection. As such, it is critical for OBE-CDMS to maximize the number of single ion signals that can be extracted in this short time. Unlike ensemble ion measurements where signals can be easily increased by simply increasing the amount of loaded analyte, for CDMS we are limited by the single ion nature of the measurements.

**Figure 3:**
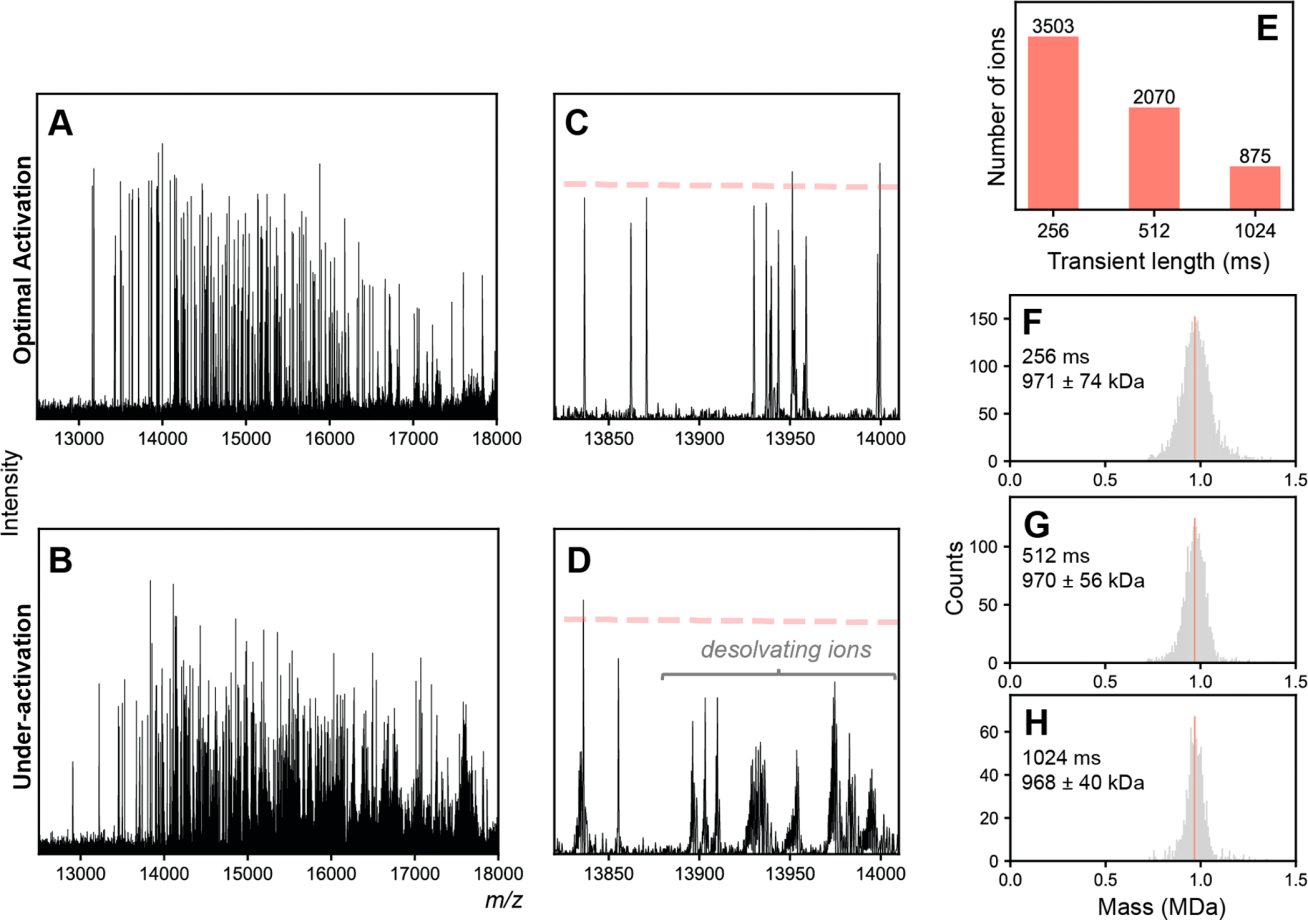
Effect of HCD and resolution settings on performance of OBE-CDMS. (A) Representative single MS scan of IgM at an optimized level of ion activation (HCD 125 V). A distinct “shelf” of single ions can be readily observed. (B) Representative single MS scan of IgM where ions are insufficiently desolvated (HCD 75 V). While some stable ions can be observed, most of the ion signals appear at lower intensity than expected, and the spectrum is unusually dense. (C,D) Magnified views of the same CDMS spectra as those in panels (A) and (B). The expected intensity of stable single ions is depicted as a dashed red line. Whereas stable, narrow ion signals can be clearly observed at optimized HCD conditions, multiple clusters of low-intensity signals are observed at insufficiently activated HCD conditions, corresponding to successive frequency shifts (*e.g*., due to desolvation) during the transient. (E) Number of stable single ions acquired from a single OBE-CDMS run as a function of the transient recording time. More ions are acquired per run at shorter transient times due to the increased scan rate. (F–H) Mass histograms of IgM obtained from OBE-CDMS at different transient times. Longer transient lengths result in narrower peak widths, and thus increased resolving power.

We found that the most impactful avenue to increasing the number of extractable detection events is to optimize the stability of the single ion signals over the recorded transient times. Normally, only signals that are both stable and persist the entire transient are kept for CDMS analysis, as only these ions maintain a linear relationship between amplitude and charge.^28^ Ions that do not follow this behavior and decay during the transients are discarded and do not contribute to the final ion statistics. While some degree of ion loss is unavoidable, the relative frequency of these events can be mitigated by optimization of experimental conditions. One parameter that is especially influential is the extent of ion activation, commonly achieved using collisional activation in the HCD cell (Figure 3A–D). This is because for large protein ions, desolvation of residual solvent molecules represents one of most prevalent mechanisms of ion signal decay during the Orbitrap acquisition.^42^ Under suboptimal collisional activation conditions where ion activation levels are insufficient to fully release residual solvent (Figure 3B,D), the majority of ion signals follow unstable trajectories and undergo frequency (*i.e*. *m*/*z*) changing events, therefore not contributing to the extracted CDMS signal. By contrast, single ion signals collected under optimized activating conditions contain a much larger number of stable ion signals that can be sequentially extracted and used in CDMS analyses (Figure 3A,C).

Besides ion activation, there are other possible avenues of increasing the ion statistics per OBE-CDMS run. Most intuitively, one would increase the scan speed (*i.e*. decrease the transient length). Unfortunately, utilizing shorter transients will necessitate a trade-off, as both *m*/*z* resolution and single ion charge accuracy in CDMS worsen with shorter detection periods.^42,43^ To determine the impact of these effects, OBE-CDMS data sets of IgM were collected using transient lengths ranging from 256 to 1024 ms (corresponding to resolution settings from 50,000 to 200,000, Figure 3E–H). While the number of detected single ions does improve roughly stepwise with the scan rate, these improved statistics come at a cost of mass resolution, as is observable in the recorded IgM peak widths. Therefore, simply increasing the scan rate does not provide a “one-size-fits-all” solution to improving the data quality of Orbitrap-based CDMS at chromatographic time scales. Nevertheless, confident Orbitrap-based CDMS mass histograms comprising more than several hundred ions could be readily obtained in a single OBE-CDMS run at all of these transient time settings (Figure 3F–H). For our studies on IgM, we chose to use the maximum transient time available on nonmodified UHMR instruments (1024 ms) to maximize the obtainable mass resolution and data quality. The Orbitrap-based CDMS mass histograms obtained in this manner (Figure 3H) are comparable in mass resolution to those previously reported of IgM using standard, static spray nano-ESI acquisition, keeping in mind that IgM is quite polydisperse due to its high glycosylation load.^10,26^

### Following the Proteolytic Degradation of IgM - Simultaneous Analyses of Co-occurring Complexes

**Figure 4:**
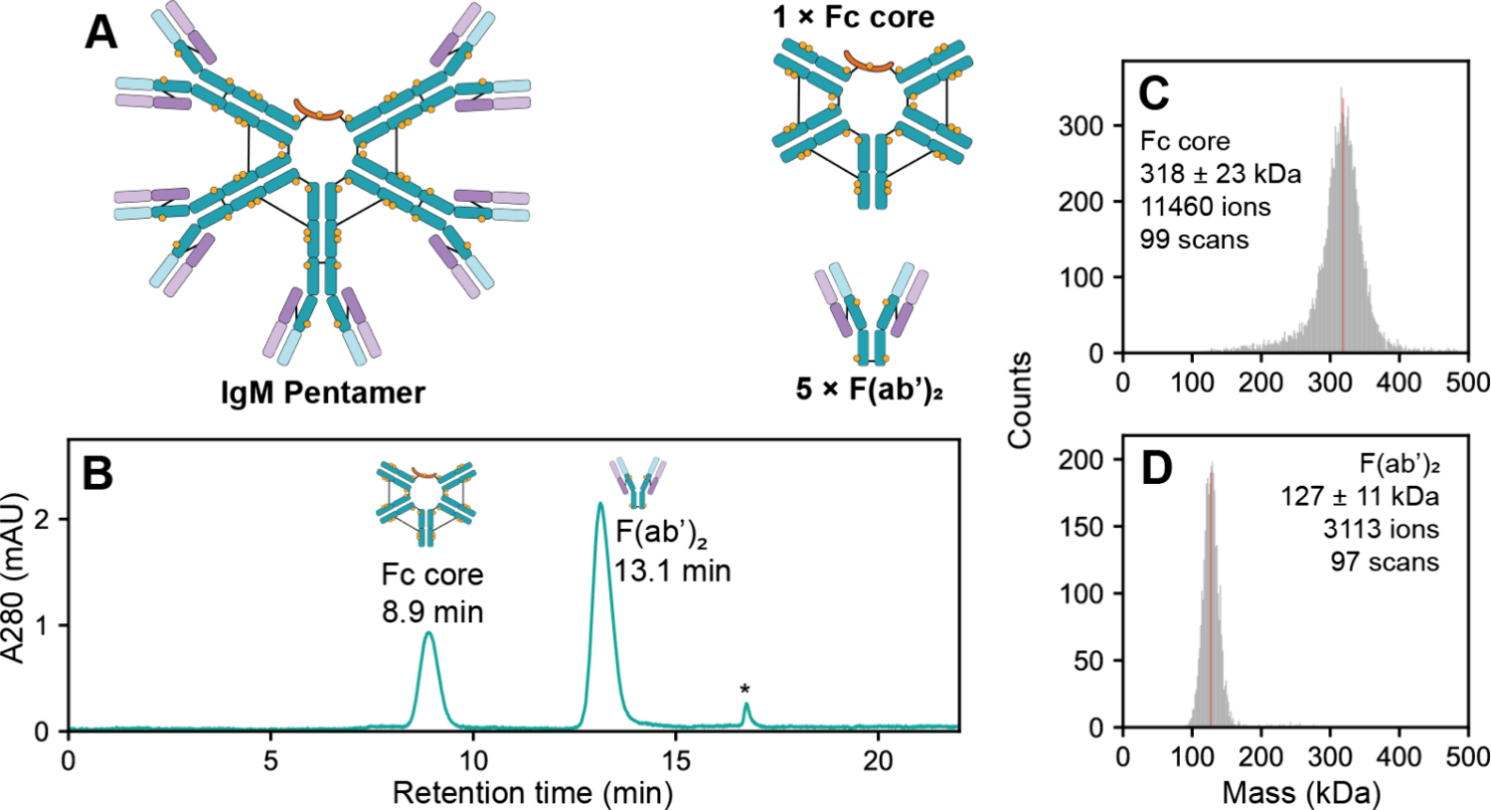
SEC-CDMS analysis of end products produced from IgMBRAZOR-digested IgM. (A) Schematic depiction of IgMBRAZOR-induced digestion of IgM. IgMBRAZOR liberates all five intact F(ab’)_2_ subunits from the IgM precursor, co-yielding the truncated pentameric Fc core. (B) UV chromatogram from SEC-CDMS of fully processed IgM. The two final products, the Fc core (29.4%) and F(ab’)_2_ (70.6%) subunits are baseline resolved, allowing MS parameters to be individually tuned for each analyte. A small peak marked by an asterisk (*) can be observed, which was found to originate from the IgMBRAZOR protease. The retention time for each species is labeled. (C–D) Mass histograms of the pentameric Fc core and F(ab’)_2_ subunits, respectively, obtained by SEC-CDMS from a single 0.5 μg injection.

The primary goal of this study is to monitor the processing of IgM by the protease IgMBRAZOR. IgMBRAZOR is an IgM-specific protease that digests human IgM at a specific site below the Cμ2 domain in the heavy chain (cleaving after amino acid 343, between ...VPDQDT/AIRVFA...) (Figure 1). Notably, to release one F(ab’)_2_ fragment the protease needs to make two cleavages (one for each Fc). Ultimately, incubation with IgMBRAZOR will liberate five F(ab’)_2_ moieties (∼130 kDa each), alongside the pentameric, J-coupled Fc core (MW ∼ 300 kDa) (Figure 4A). The short OBE column, while ideal for rapid desalting, lacks proper chromatographic separation capabilities for co-occurring protein assemblies, as all macromolecular species coelute in the dead volume. This scenario is problematic, in light of the importance of experimental parameter optimization described above, as finding a single set of MS parameters that yields good single ion behavior for all analytes simultaneously across such a wide molecular weight range can be challenging. For our case, this difficulty is further compounded because digestion of one IgM molecule is expected to generate five equivalents of F(ab’)_2_ (*i.e*. the number of molecules changes greatly during the reaction, Figure 4A), thereby running the risk of oversampling the F(ab’)_2_ and/or under-sampling the J-coupled Fc-core populations under single ion conditions, as their relative populations change over the course of the reaction.

To tackle this challenge, we switched to a longer, 30 cm SEC column which would allow us to chromatographically separate various co-occurring assemblies prior to CDMS analysis. Indeed, using this longer SEC column (hereby referred to as SEC-CDMS), the Fc core and F(ab’)_2_ subunits could be chromatographically resolved (Figure 4B). While the longer SEC column somewhat increases the runtime per measurement, the gain in chromatographic separation allows for MS parameters and ion injection times to be tuned and optimized for each analyte individually. A secondary benefit of the longer runtimes for SEC-CDMS is that chromatographic peaks also become wider in time (approximately 1 min), allowing for much more MS scans to be accommodated per injection relative to the shorter column used in OBE-CDMS. With SEC-CDMS, F(ab’)_2_ and Fc masses could be extracted (127 ± 11 and 318 ± 23 kDa, respectively, Figure 4**C, D**), in line with the predicted masses (129 and 324 kDa, respectively, assuming an average mass gain of 1.6 kDa per N-glycosylation site). Sufficient ion statistics could be obtained such that mass histograms could be extracted from a single injection.

### Real-Time Monitoring of IgM Digestion Kinetics Reveals Degradation Intermediates

We next applied SEC-CDMS to monitor the IgMBRAZOR-induced digestion of IgM over time, with the aim of analyzing intermediates and gaining insight into the mechanism of F(ab’)_2_ cleavage and release. Normally, these experimental designs can be somewhat challenging, as native MS requires volatile salts (*e.g*. ammonium acetate solution^30^) with low quantities of inorganic salts or other additives, which may not necessarily be compatible with enzymatic processes, which can require cofactors and/or specific buffer conditions. The automated SEC-CDMS coupling side-steps this limitation and ensures that the reaction is first carried out in optimal native physiological conditions using PBS buffer. The online buffer exchange into ammonium acetate then occurs right before the MS measurement, at each user-defined time point of the digestion.

**Figure 5:**
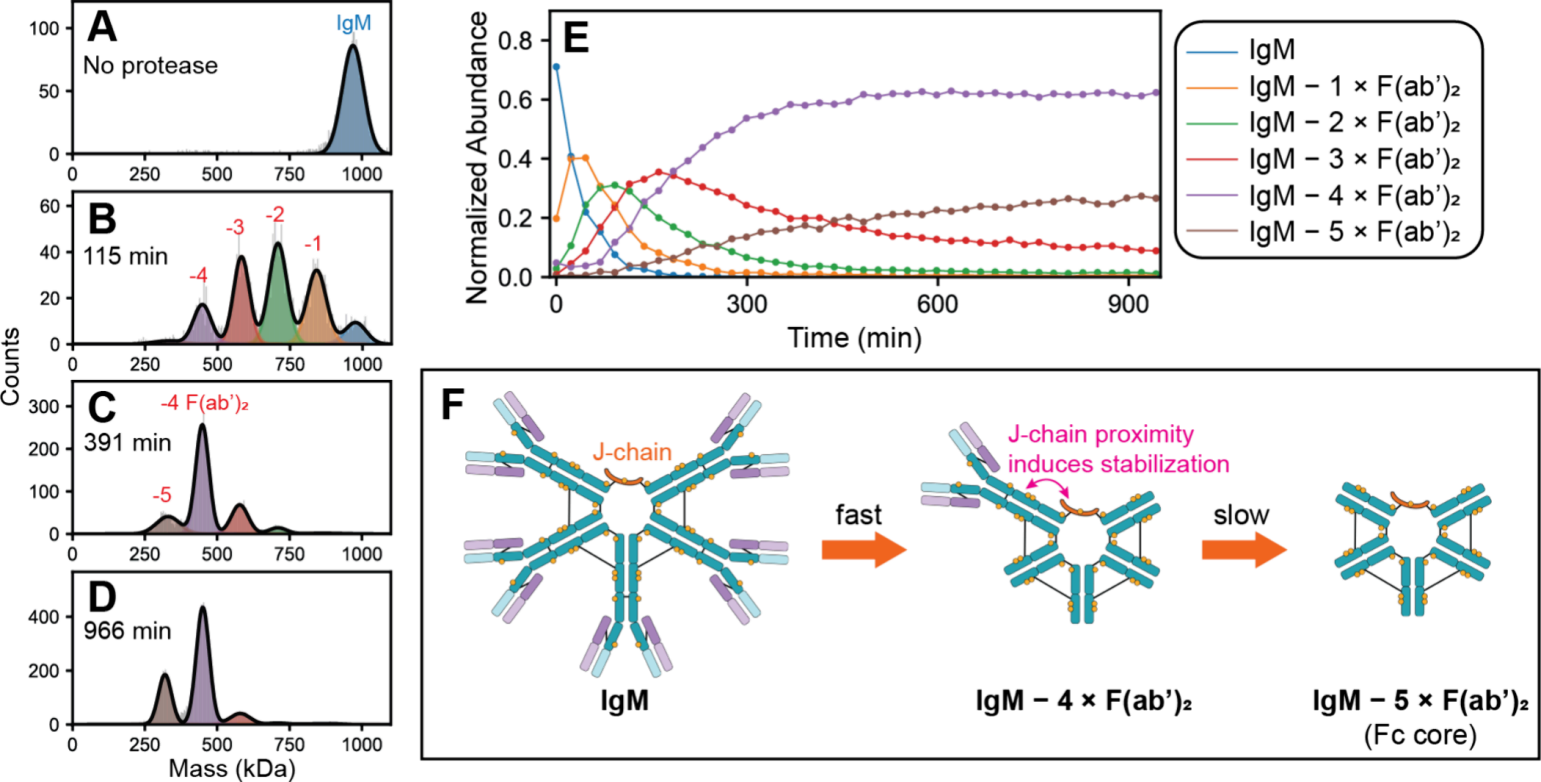
Real-time kinetics monitoring of IgM digestion using SEC-CDMS. (A–D) Representative mass histograms from SEC-CDMS depicting the conversion of intact IgM to partially processed intermediate species. Individual species resulting from processive losses of F(ab’)_2_ arms are identified by their decrease in mass. The signals for free F(ab’)_2_ are omitted for clarity. (E) Plot of individual species abundances as a function of incubation time using 1 U (0.04 U/μg IgM) of IgMBRAZOR at 7 °C. Lower temperatures and enzyme loading was deliberately used in these experiments to decrease the rate of digestion. Each time point represents an independent SEC-CDMS injection. While the first 4 F(ab’)_2_ arms are rapidly cleaved, the final fifth F(ab’)_2_ arm appears somewhat resistant to cleavage. (F) Proposed mechanism of IgM digestion by IgMBRAZOR. Cleavage of the first four F(ab’)_2_ moieties occurs quickly, leading to rapid formation of the [IgM – 4 × F(ab’)_2_] species. The final fifth F(ab’)_2_ is conformationally restricted and less accessible to IgMBRAZOR, relative to the other arms, likely due to proximity to the interacting J-chain. Prolonged incubation will eventually cleave the final F(ab’)_2_, yielding the fully processed Fc core (Figure S1).

We first confirmed, by using SDS-PAGE, that IgMBRAZOR completely and rapidly digests IgM under normal conditions (see Experimental Section, Figure S1). When substantially decreasing the protease concentration (*i.e*. ≪1 U), multiple bands corresponding to intermediate digestion products appear on the gels (Figure S1), but the low resolution of the gel prevents further detailed interpretation. However, using SEC-CDMS we could monitor the IgMBRAZOR-induced reaction, directly identifying and quantifying each unique digestion product over time (Figure 5). To render the IgMBRAZOR reaction amenable to the time scale of a SEC-CDMS experiment (∼23 min per measurement), we decreased both the protease concentration and solution temperature to slow down the conversion process (see Experimental Section). Monitoring of the reaction by UV alone readily distinguishes the formation of liberated F(ab’)_2_ appearing at later retention times, but identification of partially processed IgM intermediates remains difficult due to their poor separation by SEC, appearing as a broad, generally unresolved chromatographic feature (Figure S2).

By contrast, several clearly resolved new species of different masses could be readily resolved and mass analyzed using SEC-CDMS (Figure 5B–D). We identified these intermediates as IgM molecules missing one (842 ± 34 kDa), two (708 ± 31 kDa), three (582 ± 28 kDa), and four (448 ± 31 kDa) arms (Figure 5B). Considering the high degree of symmetry within the IgM and identical amino acid sequences of each F(ab’)_2_ subunit, one would expect *a priori* that IgMBRAZOR would not exhibit preferential cleavage toward any of the five F(ab’)_2_ present on the pentameric IgM molecule. Surprisingly, this is not what is observed (Figure 5E). While the removal of the first four F(ab’)_2_ subunits appears to occur rapidly, the digestion drastically slows down when releasing the last F(ab’)_2_ arm, as evidenced by the accumulation of the [IgM – 4 × F(ab’)_2_] species even at extended incubation times (Figure 5C,D). Kinetic modeling of data in Figure 5E using a simple sequential digestion model suggests that the final step, *i.e*. formation of [IgM – 5 × F(ab’)_2_], is more than an order of magnitude slower than the preceding steps (Figure S3, Table S2). We confirmed that this “stalling” is also observed at increased protease concentrations (Figure S4), although it is crucial to stress that the final fifth F(ab’)_2_ is eventually cleaved off to yield the final Fc core (Figure 4B, Figure S1).

Given that the five F(ab’)_2_ arms of IgM are identical in sequence, why would the final F(ab’)_2_ subunit show differential kinetics? A putative answer to this question may come from recent cryo-EM structures of full-length pentameric IgM, suggesting that not all five F(ab’)_2_ arms are structurally equivalent.^44,45^ Normally, Fab domains of antibodies are difficult to visualize by structural methods due to the intrinsic flexibility and resulting conformational heterogeneity.^46^ However, a recent structure by Chen et al. revealed that one of the five F(ab’)_2_ arms (connected to the J-chain, Figure 5F) in IgM is substantially more rigid and conformationally restricted than the other four.^44^ We postulate that this J-chain induced, symmetry-breaking conformational inflexibility could impede the digestion of this specific F(ab’)_2_ arm, leading to the observed stalled digestion kinetics for the final fifth F(ab’)_2_ subunit. The other F(ab’)_2_ moieties that are not stabilized by the J-chain can sample much larger bending angles,^44^ and so conformational flexibility likely make those four arms more accessible to IgMBRAZOR (Figure 5F).

To seek validation for this hypothesis, we considered that if the stalled kinetics of the fifth F(ab’)_2_ is indeed driven by the proximity and connection to the J-chain, then this should not be observed if the J-chain is absent. Therefore, we performed identical IgMBRAZOR digestion experiments on an identical recombinant IgM construct, albeit lacking the J-chain (hereby referred to as IgM_ΔJ, Figure S5). Unlike its native, J-chain containing counterpart, which is exclusively pentameric, recombinant IgM lacking the J-chain tend to form a mixture of oligomeric species.^26,47,48^ Upon addition of IgMBRAZOR, this additional heterogeneity initially yields an unresolved mixture of partially digested species arising from these different oligomers (Figure S5F). However, when directly comparing the digestion of IgM and IgM_ΔJ at intermediate/late reaction times, no evidence of digestion stalling is observed for IgM_ΔJ, as the fully processed Fc core is reached rapidly and without accumulation of partially digested intermediates (Figure S5G,H). These data support the hypothesis that the presence of the J-chain influences the structure and symmetry of IgM and affects its digestion kinetics, although further work may be needed to confirm the exact identity of the stalled fifth F(ab’)_2_ subunit. Nevertheless, our data clearly demonstrate the utility of SEC-CDMS in investigating these complex kinetic processes.

## Conclusions

Here we demonstrate that SEC-CDMS enables the mass determination of heterogeneous high mass macromolecules, in a single run, as exemplified here by the analysis of IgM and its proteolytic cleavage products. We evaluated key parameters (HCD, resolution, injection times) that need to be optimized for successful SEC-CDMS experiments. Nonideal ion behaviors can be mitigated by increasing the HCD values, reducing the resolution, or avoiding higher flow rates that typically come with poor desolvation during the ESI process. We also showed that using a fixed injection time does not necessarily increase ion overlap in CDMS of heterogeneous macromolecules, and so controlling the ion flux along the elution for these analyte types is not essential to successfully perform LC-CDMS.^39^ Here, we obtained accurate masses of several co-occurring macromolecules in a single run, but ion statistics could also be further improved by summing multiple runs, as previously proposed.^35^ Short OBE columns are the go-to option to rapidly assess sample heterogeneity and assign masses. For quantification purposes or more complex mixtures, longer columns are beneficial to set optimized MS parameters for each eluted species and ensure proper characterization of lower abundant species. In all cases, coupling SEC to CDMS substantially reduces analysis time by automating the upstream buffer exchange step.

By monitoring digestion kinetics of IgM into F(ab’)_2_ and Fc subunits, strikingly different behaviors of the five F(ab’)_2_ arms were unveiled, with the first four cleaved off efficiently, and the final, fifth arm more resistant to cleavage. In contrast, for IgM_ΔJ, we observed facile cleavage of all F(ab’)_2_ arms. Therefore, we hypothesize that the fifth F(ab’)_2_ arm is distinct in IgM due to its proximity and unique interaction with the J-chain, making it less accessible to IgMBRAZOR cleavage. Taking this one step further, it is likely that the binding sites of the complement component 1q (C1q) are less exposed in IgM in part because of the asymmetric behavior of the stabilized F(ab’)_2_ toward antigen binding, whereas the complement deposition in IgM_ΔJ may be more symmetric.^44 ,45^ Based on literature and the work presented here, it is clear that J-chain incorporation into IgM has a profound effect on its structure, function, and stability. J-chain incorporation also affects the overall symmetry of the IgM molecule. The starfish-like IgM_ΔJ exhibits C5 symmetry (Figure 1B) but becomes pseudo-C2 when the J-chain is incorporated in IgM (Figure 1C). Evidently, this pseudo-C2 symmetry may in reality be closer to C1, as the J-chain is not symmetrically placed, does not interact similarly with the two IgM protomers it links to, and seemingly influences *e.g*. its interaction with proteases such as IgMBRAZOR in an asymmetric manner.

Moving forward, SEC-CDMS (and even other nondenaturing LCs), could be applied for the characterization of other biologically relevant samples. Native SEC-MS is already well integrated into R&D pipelines of biopharmaceutical companies, and by implementing SEC-CDMS the range of applications for therapeutic products might be expanded. Indeed, with the latest generation of SEC columns, it now becomes possible to tackle increasingly complex analytes of different nature and size, including membrane proteins,^40^ glycoproteins,^35^ plasmid DNAs,^49^ mRNA, or adeno-associated viruses.

## Supporting Information Available

Tabulations of experimental LC and MS parameters; SDS-PAGE analysis of IgMBRAZOR digestion; additional results of IgMBRAZOR digestion kinetics of IgM constructs (PDF)

## Dedication

Dedicated to Dame Carol Robinson, whom A.J.R.H. considers as a longtime friend and excellent colleague, in honor of her 2023 ASMS Fenn Distinguished Contribution award.
