# Supplementary material for: Not All Arms of IgM Are Equal: Following Hinge-Directed Cleavage by Online Native SEC-Orbitrap-Based CDMS
Source: J Am Soc Mass Spectrom. 2024 May 20;35(6):1320–9. doi: 10.1021/jasms.4c00094 (PMC11157650; doi:10.1021/jasms.4c00094)
Supplement: Supplementary file 1 — js4c00094_si_001.pdf [file js4c00094_si_001.pdf]

SUPPORTING INFORMATION

Not All Arms of IgM Are Equal: Following Hinge-Directed Cleavage by Online Native SEC-Orbitrap-Based CDMS

Victor Yin[1,2,#], Evolène Deslignière[1,2,#], Nadia Mokiem[1,2], Inge Gazi[1,2], Rolf Lood[3], Carla J.C. de Haas[4], Suzan H.M. Rooijakkers[4], Albert J.R. Heck[1,2,*]

[1] Biomolecular Mass Spectrometry and Proteomics, Bijvoet Center for Biomolecular Research and Utrecht Institute for Pharmaceutical Sciences, Utrecht University, Padualaan 8, 3584 CH, Utrecht, The Netherlands

[2] Netherlands Proteomics Center, Padualaan 8, 3584 CH, Utrecht, The Netherlands

[3] Genovis AB, Scheelevägen 2, 223 63 Lund, Sweden

[4] Department of Medical Microbiology, University Medical Center Utrecht, Utrecht University, 3584 CX, Utrecht, the Netherlands

**Corresponding author: a.j.r.heck@uu.nl*

Table S1. Tabulation of LC and MS experimental parameters used for SEC-CDMS experiments using the two different columns.

		OBE-CDMS	Analytical SEC-CDMS
LC parameters	Injected quantity	0.5 µg	
	Injected volume	2 µL	
	Flow rates	100 µL/min	300 µL/min (0-5.4 min)<br>100 µL/min (5.5-15 min)<br>300 µL/min (15.1-22 min)
	Total gradient time (min)	5	22
MS parameters	Polarity	Positive	
	Capillary voltage (kV)	2.7	
	Capillary temperature (°C)	250	
	S-lens RF level (%)	200	
	m/z range	5,000-20,000	5,000-20,000 for IgM and partial complexes<br><br>2,500-10,000 for F(ab')$_2$
	Ion injection time (ms)	c.a. 100	c.a. 100 for IgM<br>c.a. 5 - 50 for F(ab')$_2$
	Transient time (ms)	256, 512 or 1024	1024
	Microscans	1	
	Noise threshold	0	
	Source DC offset (V)	21	
	In-source trapping	Off	
	HCD voltage (V)	125	
	Trap gas setting	4.5	
	UHV readout (1e-10 mbar)	3	
	Collision gas	Nitrogen	
	Injection flatapole (V)	7	
	Inter-flatapole lens (V)	7	
	Bent flatapole (V)	7	
	Ion transfer target	High m/z	
	Detector optimization	High m/z	

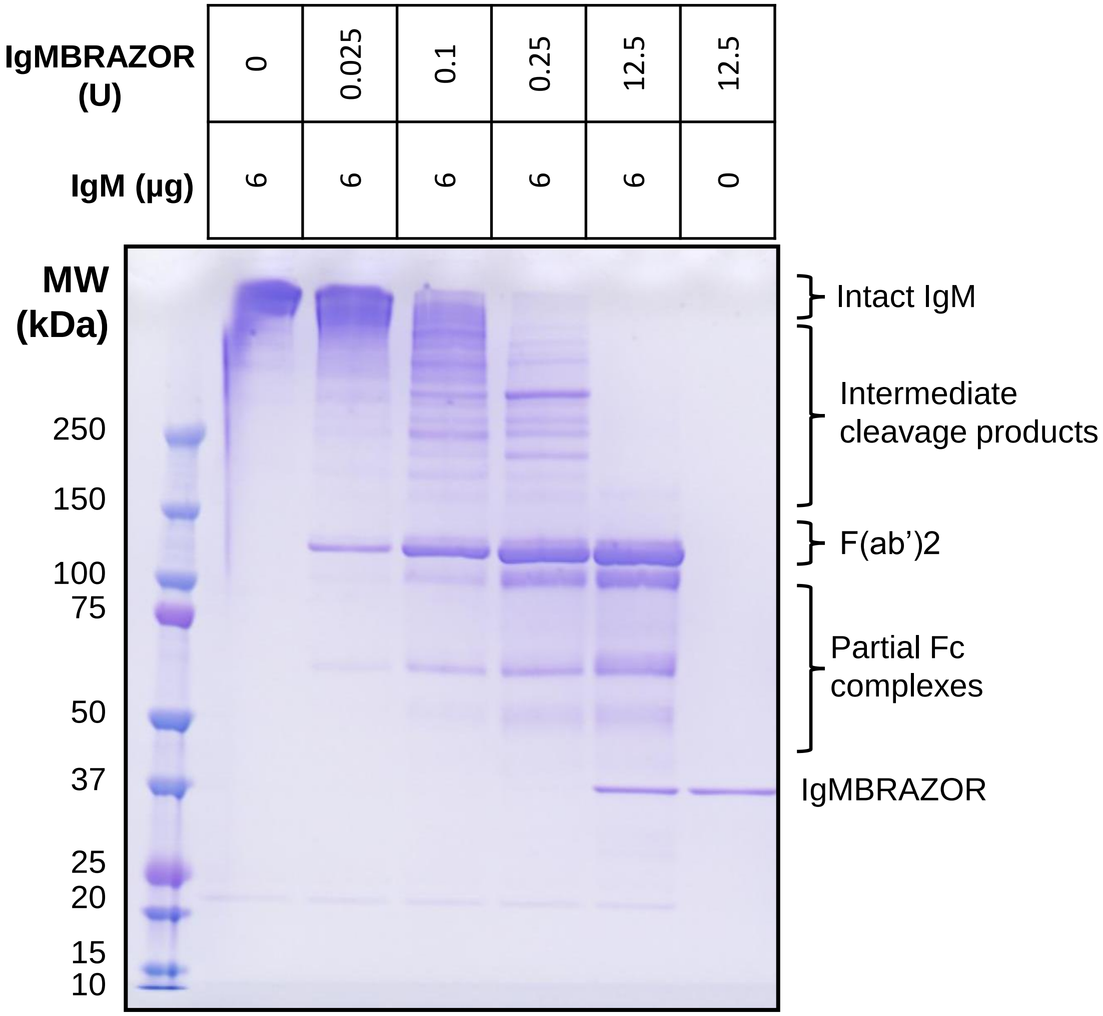

Figure S1: Monitoring IgM digestion by IgMBRAZOR by SDS-PAGE. Non-reducing SDS-PAGE analysis of IgM following incubation with IgMBRAZOR. New progressive bands at lower molecular weight (between 100 and 800 kDa) are formed upon incubation, confirming that IgMBRAZOR proteolytically processes IgM in distinct steps. Higher concentrations of IgMBRAZOR yield a greater and faster degree of conversion to F(ab')$_2$. At sufficiently high concentrations of IgMBRAZOR, IgM is fully converted. For each lane, IgM was digested for 30 minutes at ambient temperature.

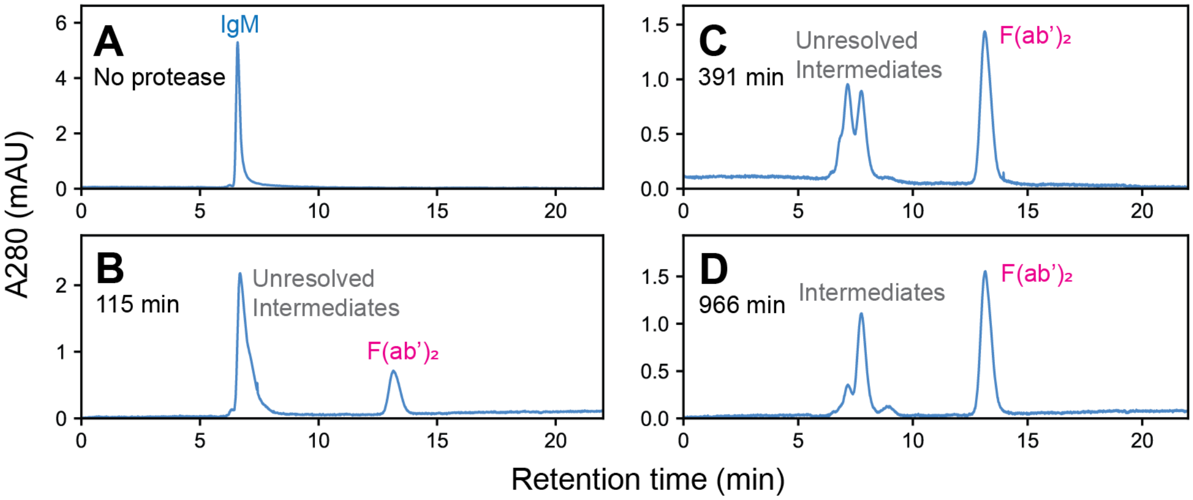

Figure S2: Representative UV chromatograms of IgMBRAZOR-processed IgM at different time points. (A) Control without protease. (B) 115 minutes. (C) 391 minutes. (D) 966 minutes. The time points shown are identical to those depicted in Figure 5A-D. Digestion was performed using 1 U (0.04 U/µg IgM) of IgMBRAZOR at 7 °C. While the formation of $F(ab')_2$ can be easily followed as a function of incubation time, the direct monitoring of the various partially digested intermediates is less straightforward to their poor resolution by SEC retention time.

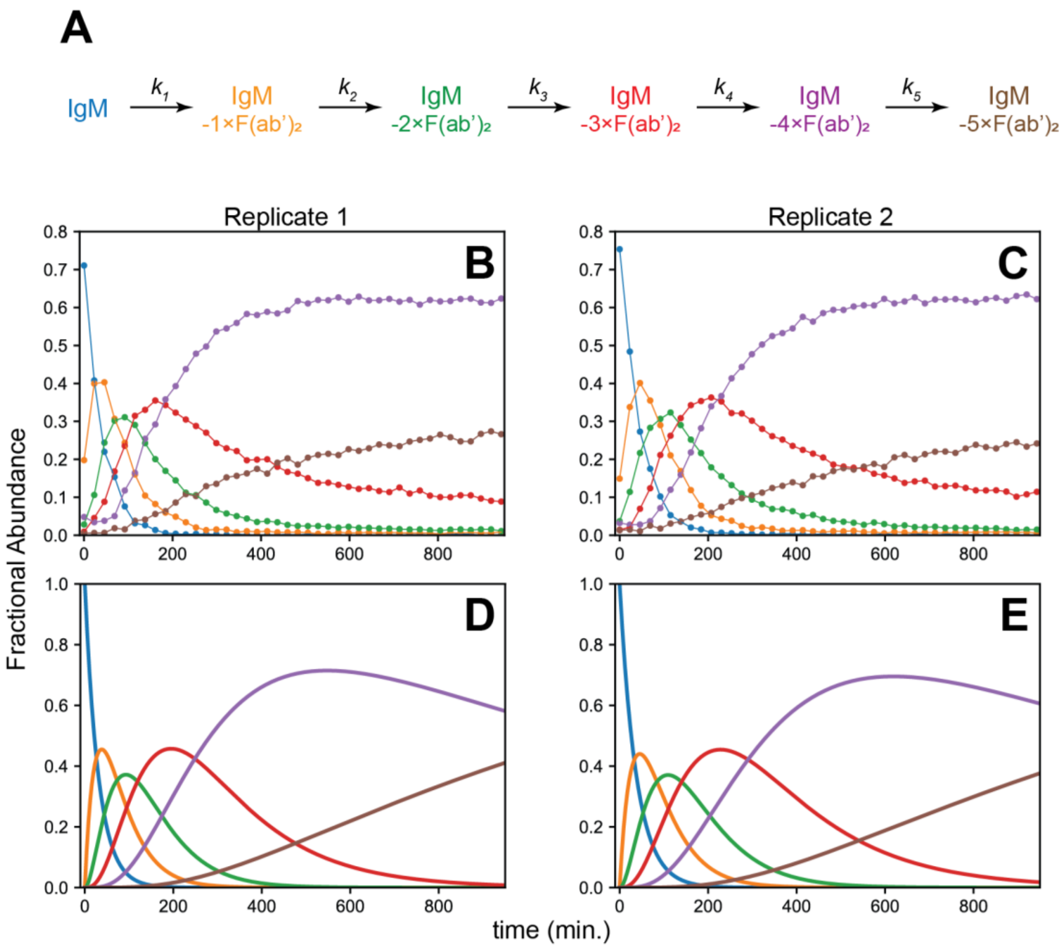

Figure S3: Kinetic modeling of IgM digestion by 1 U IgMBRAZOR at 7 °C. (A) Schematic diagram of the minimalistic kinetic model utilized for fitting. The formation of each digested IgM species is described by a single first order rate constant, yielding 5 independent rate constants (k_1 – k_5) for the overall process. (B-C) Real-time kinetic monitoring of two independent experimental replicates of the IgM digestion process. The data in panel B is also depicted in Figure 5E. (D) Calculated kinetic profile obtained by fitting the experimental data of panel B. (E) Calculated kinetic profile obtained by fitting the experimental data of panel C.

Table S2: Tabulation of average fitted rate constants for IgM digestion by 1 U IgMBRAZOR at 7 °C. The depicted values are the average and standard deviations between all replicates.

	Fitted rate constants (U^{-1} min^{-1})		
k_1	2.94E-02	±	0.43E-02
k_2	1.93E-02	±	0.18E-02
k_3	1.39E-02	±	0.15E-02
k_4	5.86E-03	±	0.59E-03
k_5	8.25E-04	±	0.18E-04

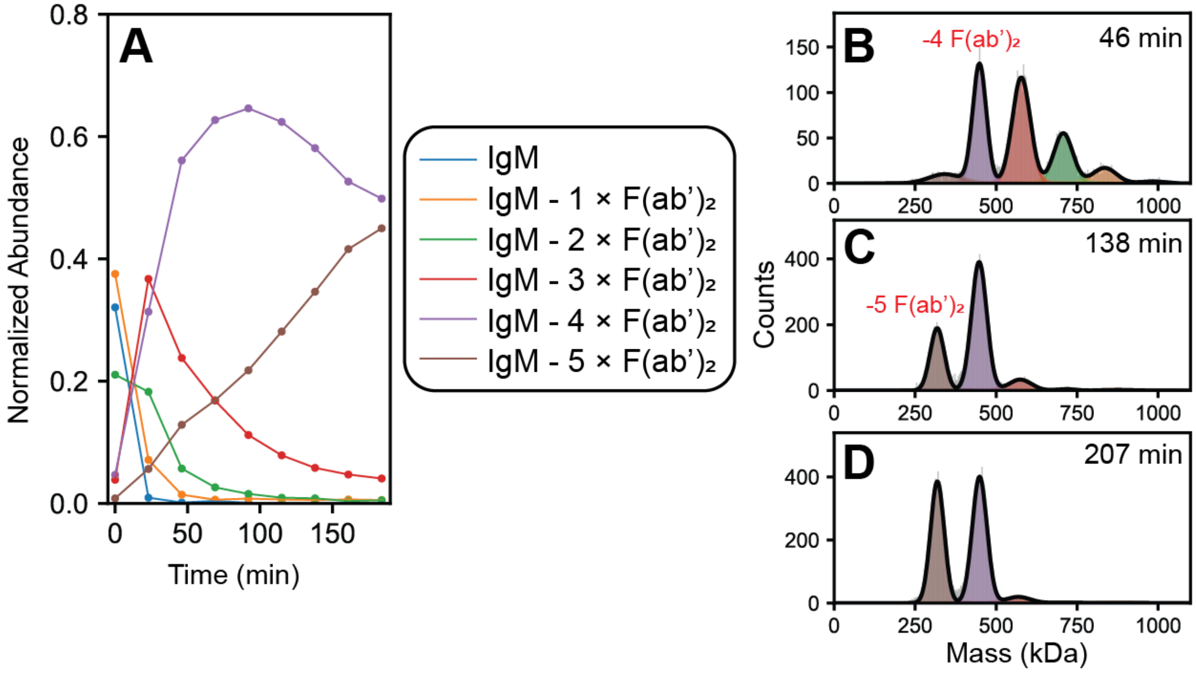

Figure S4: Real-time digestion kinetics of IgM at increased IgMBRAZOR load (5 U, or 0.2 U/µg IgM) and 7 °C measured by SEC-CDMS. (A) Plot of individual species abundances as a function of IgMBRAZOR incubation time. (B-D) Representative mass histograms from SEC-CDMS depicting the processing of IgM by IgMBRAZOR. In comparison to the data in Figure 5, the conversion at higher IgMBRAZOR concentrations happens substantially faster. At later time points, a major peak corresponding to formation of the final Fc core product (*i.e.* IgM – 5×F(ab')$_2$) is readily observed.

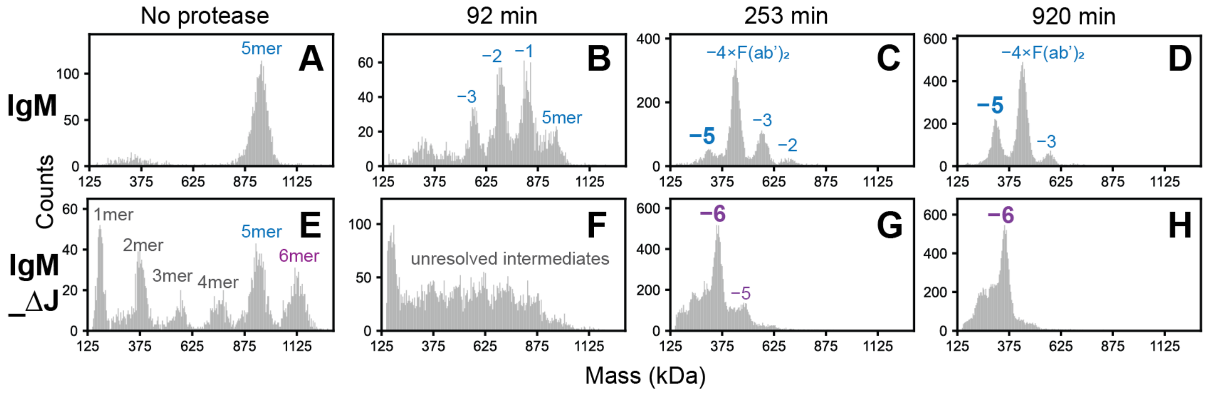

Figure S5: Real-time digestion kinetics of IgM lacking the J-chain. (A-D): IgM containing the J-chain (IgM) as a control. (E-H): IgM expressed without the J-chain (IgM_ΔJ). In all experiments 1 U IgMBRAZOR (0.04 U/µg IgM) was used and reacted at 7 °C. In the absence of J-chain, recombinant IgM forms a mixture of primarily tetra-, penta- and hexamers (labeled). Each column depicts an equivalent degree of digestion by IgMBRAZOR. Major species are labeled according to the number of missing $F(ab')_2$ subunits, and coloured depending on the parent oligomer: pentamer (blue), or hexamer (purple). The final product corresponding to a fully processed $(Fc)_n$ core is also labeled in bold for further emphasis. Unlike the regular IgM, for IgM lacking the J-chain, no evidence of stalled digestion, *i.e.* accumulation of Fc core species possessing one remaining $F(ab')_2$, is observed. The major species is the Fc core with all $F(ab')_2$ moieties proteolytically removed.
